# Quantitative electroencephalographic biomarker of pharmacological treatment response in patients with anxiety disorder: a retrospective study

**DOI:** 10.1038/s41598-023-30994-1

**Published:** 2023-03-07

**Authors:** Jun Byeon, Jung Yeon Moon, Se Ryoung Je, So Hyun Park, Jun Won Kim

**Affiliations:** 1 Department of Psychiatry, Daegu Catholic University School of Medicine, Daegu, Republic of Korea; 2 Department of Psychiatry, Ewha Womans University Mokdong Hospital, Ewha Womans University College of Medicine, Seoul, Republic of Korea

**Keywords:** Anxiety, Electroencephalography - EEG, Predictive markers

## Abstract

This study aimed to investigate the effectiveness of a quantitative electroencephalography (qEEG) biomarker in predicting the response to pharmacological treatment in patients with anxiety disorder. A total of 86 patients were diagnosed with anxiety disorder according to the Diagnostic and Statistical Manual of Mental Disorders 5th edition, and subsequently treated with antidepressants. After 8–12 weeks, the participants were divided into treatment-resistant (TRS) and treatment-response (TRP) groups based on their Clinical Global Impressions-Severity (CGI-S) scores. We obtained the absolute-EEG measurements for 19-channels and analyzed qEEG findings according to the frequency range: delta, theta, alpha, and beta. The beta-wave was subdivided into low-beta, beta, and high-beta waves. The theta-beta ratio (TBR) was calculated, and an analysis of covariance was performed. Of the 86 patients with anxiety disorder, 56 patients (65%) were classified in the TRS group. The TRS and TRP groups did not differ in terms of age, sex, or medication-dosage. However, the baseline CGI-S was higher in the TRP group. After calibration by covariates, the TRP group showed higher beta-waves in T3 and T4, and a lower TBR, especially in T3 and T4, than the TRS group. These results indicate that patients with a lower TBR and higher beta and high-beta waves in T3 and T4 are more likely to respond to medication.

## Introduction

Anxiety disorders, are the most common form of psychiatric disease worldwide, with a 21.3% 1-year prevalence rate and a 33.7% lifetime prevalence^1^. They include panic disorder, generalized anxiety disorder, social anxiety disorder, specific phobia, and separation anxiety disorder, and are often comorbid with other psychiatric diseases, necessitating prolonged treatment periods^1^. The most common pharmacological agents used for treating anxiety disorders are selective serotonin reuptake inhibitors (SSRIs) and serotonin-norepinephrine reuptake inhibitors (SNRIs)^2^. However, approximately 50% of adults and 40% of children and adolescents with anxiety disorders are unresponsive to pharmacological treatment^3^. The reason for the poor treatment response depends on each individuals’ underlying pathophysiology; however the underlying mechanism is unknown and involves multiple neurotransmitters^4^. For this reason, several attempts have been made to evaluate the treatment response of patients with anxiety disorders, especially through development of predictors of the treatment response^5^.

To date, functional magnetic resonance imaging (fMRI)^6,7^, magnetic resonance spectroscopy (MRS)^8,9^, heart rate variability (HRV)^10^, brain-derived neurotrophic factor (BDNF)^11^, and 5-HT2A gene polymorphisms have been studied as predictors. Among these, quantitative electroencephalography (qEEG) data, which can serve as a biological marker of brain function by providing information about the electrophysiological activity of the brain, can be obtained without invasive approaches such as needle or radiation exposure, and can be acquired faster than other diagnostic tools. Due to these advantages, qEEG studies on the treatment response in patients with depressive disorder are being actively conducted. However, there have been considerably fewer EEG studies on the treatment response in patients with anxiety disorders.

Currently, the most prominent qEEG finding in anxiety disorders is an increase in beta waves in the temporal lobe^12^. Beta waves are the basic human arousal waveforms associated with attention and reflect significant levels of local metabolic activity, particularly in the cortex^13^. These waves can be roughly divided into low-beta waves (12–15 Hz), beta waves (15–25 Hz), and high-beta waves (25–30 Hz)^14^. However, high-beta waves are particularly associated with individual fear, anxiety, and hyperarousal^12,15^. Moreover, a higher theta-beta ratio (TBR) is associated with increased anxiety^16^. This pattern occurs during thalamocortical dysrhythmia (TCD) and is associated with attention problems and overreaction to fear^17^.

In this study, we aimed to predict the response to pharmacological treatment for anxiety disorders by considering the characteristics of the various qEEG findings described above.

## Results

### Demographic characteristics

The study included 56 patients in the TRS group and 30 patients in the TRP group. There were no significant differences in age; sex; history of prescription drugs; or State-Trait Anxiety Inventory (STAI-S, STAI-T), and Beck Depression Inventory (BDI) scores between the two groups. However, the initial CGI-S score was significantly higher in the TRP group (*p* = 0.043). Most subscales of the Symptom Checklist-90-Revised (SCL-90-R) did not differ significantly between the two groups, but the TRS group showed a significantly higher score on the HOS scale (*p* = 0.006) (Table 1).

**Table 1:** Clinical characteristics in each group.

Characteristics	Treatment resistant anxiety disorder (n = 56)	Treatment response anxiety disorder (n = 30)	t-value	*P*-value
Sex, n(%)				0.983
Male	26(46.4%)	14(46.6%)		
Female	30(53.6%)	16(53.4%)		
Age	50.39 ± 19.23	48.80 ± 15.43	0.391	0.697
CGI-S	4.73 ± 0.73	5.10 ± 0.90	− 2.057	0.043*
AD equivalent	20.32 ± 9.23	23.48 ± 10.3	− 1.452	0.150
BZD equivalent	7.05 ± 3.88	8.1 ± 5.98	− 1.045	0.299
STAI-S	56.39 ± 11.88	54.18 ± 11.33	0.749	0.456
STAI-T	56.11 ± 12.08	54 ± 12.52	0.686	0.495
BDI	26.64 ± 11.90	23.86 ± 10.11	0.966	0.337
SCL-90-R				
SOM	55.143 ± 11.444	56.682 ± 11.193	− 0.538	0.592
O-C	59.054 ± 10.346	56.864 ± 8.38	0.884	0.379
I-S	58.482 ± 11.014	53 ± 10.668	1.995	0.050
DEP	61.964 ± 10.502	58.591 ± 8.573	1.340	0.184
ANX	60.161 ± 10.908	61.682 ± 9.692	− 0.571	0.570
HOS	57.518 ± 11.233	49.773 ± 9.981	2.824	0.006*
PHOB	58.732 ± 10.335	57.364 ± 12.67	0.493	0.623
PAR	55.964 ± 11.654	50.864 ± 11.671	1.739	0.086
PSY	55.875 ± 9.029	53.545 ± 8.14	1.053	0.296
GSI	59.321 ± 9.416	57.272 ± 7.881	0.903	0.369

*p < 0.05. *CGI-S* clinical global impression-severity, *AD*: antidepressant, *BZD* benzodiazepine, *STAI-S* state-trait anxiety inventory-state, *STAI-T* state-trait anxiety inventory-trait, *BDI* beck depression inventory, *SCL-90-R* symptom checklist-90-revised, *SOM* somatization, *O–C* obsessive–compulsive, *I-S* interpersonal sensitivity, *DEP* depression, *ANX* anxiety, *HOS* hostility, *PHOB* phobic anxiety, *PAR* paranoid ideation, *PSY* psychoticism, *GSI* general symptom index.

Significant values are in bold.

### Comparison of qEEG findings

**Figure 1:**
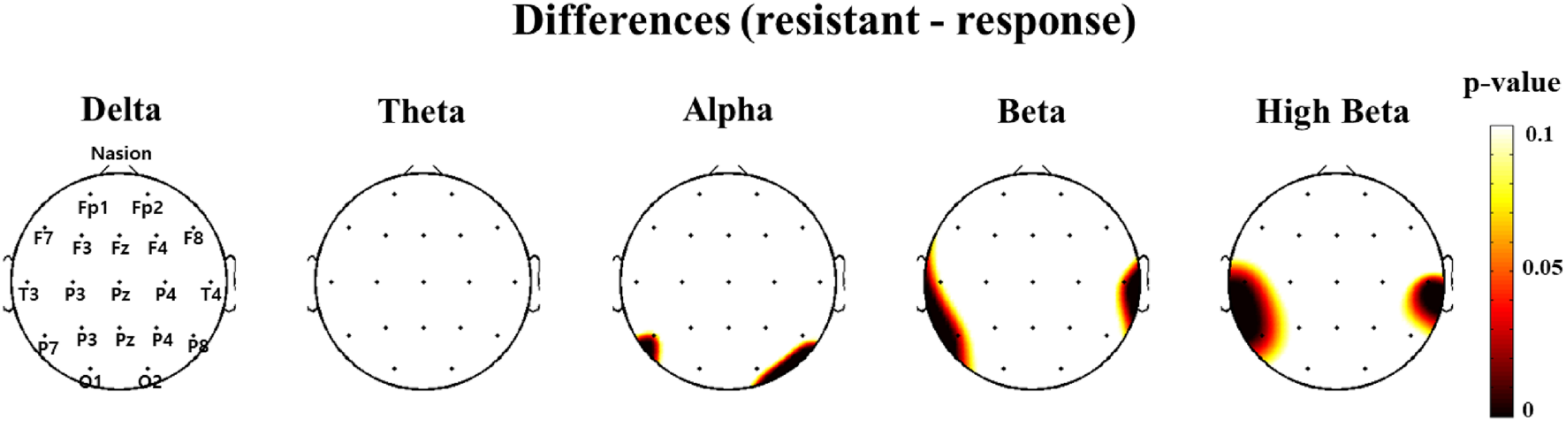
Topographic maps between channel in each frequency. The topographic maps represent the probability of analysis of covariance (ANCOVA) between two groups. A colored area means an increase of difference in absolute powers. In beta wave and high-beta wave, significant differences showed in T3 and T4.

After controlling for covariates, analysis of covariance (ANCOVA) was used to compare the absolute power average values for the total electrode sites between the two groups and for each frequency band. Although no significant differences were observed in the delta, theta, and alpha waves, the T3 (*p* = 0.030), T4 (*p* = 0.003), and T5 (*p* = 0.008) regions of the beta wave showed significant intergroup differences (Table 2). When ANCOVA was performed by subdividing the beta region into low-beta wave, beta wave, and high-beta wave, no significant intergroup differences were observed in low-beta or beta waves, but the T3 (*p* = 0.043) and T4 (*p* = 0.019) regions of the high-beta waves showed significant differences (Table 2). When the *p*-values of the beta and high-beta waves were shown in a topographical map, the findings for both temporal lobes were confirmed to be significant (Fig. 1).

**Table 2:** ANCOVA result of resting electroencephalogram in each group in beta wave.

EEG channel	Treatment resistant anxiety disorder (n = 56) (uV^2^)	Treatment response anxiety disorder (n = 30) (uV^2^)	t-value	*P*-value
Beta wave				
FP1	7.031 ± 4.204	8.314 ± 4.583	2.150	0.147
FP2	7.318 ± 3.98	8.391 ± 4.807	1.756	0.189
F7	5.728 ± 3.245	6.741 ± 3.852	2.203	0.142
F3	11.41 ± 8.806	12.255 ± 8.452	0.346	0.558
Fz	11.023 ± 8.002	12.1 ± 8.302	0.657	0.420
F4	12.078 ± 9.153	12.952 ± 9.421	0.382	0.538
F8	6.487 ± 3.369	7.304 ± 4.614	1.452	0.232
** T3**	**6.123 ± 3.268**	**7.784 ± 5.083**	**4.878**	0.030*
C3	12.212 ± 9.163	13.992 ± 10.879	0.990	0.323
Cz	14.69 ± 13.29	15.917 ± 11.034	0.342	0.561
C4	12.575 ± 8.97	14.472 ± 11.924	1.072	0.304
** T4**	**5.551 ± 2.771**	**8.052 ± 5.404**	**9.412**	0.003*
** T5**	**7.001 ± 3.912**	**10.438 ± 8.637**	**7.539**	0.008*
P3	12.663 ± 9.455	15.769 ± 12.271	2.102	0.152
Pz	13.221 ± 9.726	16.405 ± 12.817	1.984	0.163
P4	12.631 ± 8.642	15.421 ± 13.431	1.697	0.197
T6	7.288 ± 4.403	9.429 ± 7.999	2.883	0.094
O1	11.211 ± 8.173	14.208 ± 10.371	2.182	0.144
O2	11.701 ± 7.824	15.192 ± 15.404	1.993	0.162
High beta wave				
FP1	17.440 ± 6.028	17.882 ± 7.558	0.138	0.712
FP2	17.540 ± 5.871	17.959 ± 7.406	0.104	0.748
F7	17.261 ± 6.102	18.129 ± 7.262	0.422	0.518
F3	20.900 ± 8.076	22.143 ± 8.801	0.527	0.470
Fz	19.382 ± 7.591	20.58 ± 8.984	0.516	0.475
F4	20.762 ± 7.877	22.135 ± 9.354	0.665	0.418
F8	18.446 ± 5.955	18.975 ± 7.487	0.214	0.645
**T3**	**22.992 ± 6.720**	**26.338 ± 8.513**	**4.238**	0.043*
C3	22.969 ± 9.015	25.629 ± 10.026	1.713	0.195
Cz	20.722 ± 8.387	22.445 ± 9.628	0.867	0.355
C4	23.466 ± 9.067	25.522 ± 10.667	1.030	0.314
**T4**	**22.230 ± 7.043**	**26.734 ± 9.490**	**5.765**	0.019*
T5	21.986 ± 7.326	24.677 ± 9.718	1.901	0.172
P3	23.371 ± 8.828	26.543 ± 9.888	2.225	0.140
Pz	21.170 ± 7.981	24.639 ± 9.014	3.139	0.081
P4	22.928 ± 8.641	25.874 ± 9.500	1.874	0.175
T6	20.894 ± 7.782	22.942 ± 8.959	0.999	0.321
O1	21.084 ± 7.794	22.803 ± 8.659	0.748	0.390
O2	21.099 ± 8.577	22.053 ± 8.387	0.274	0.603

*p < 0.05. *EEG* electroencephalogram.

Significant values are in bold.

### Comparison of TBR

**Figure 2:**
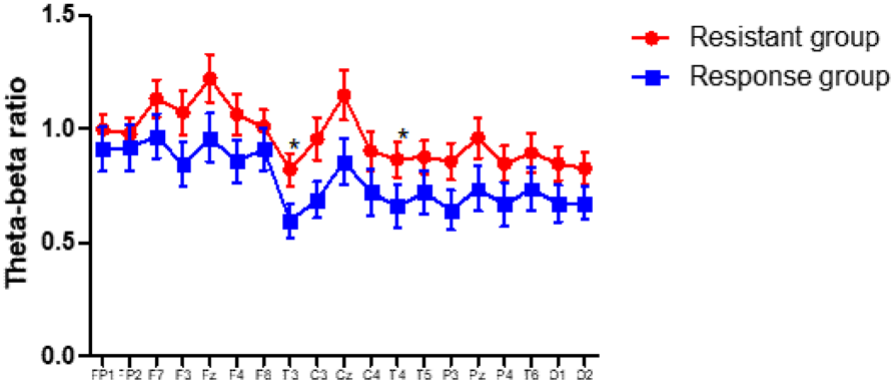
Theta-beta ratio between channel in each group. Figure 2 shows the difference in Theta-Beta ratio (TBR) between two groups for each channel. Each graph moves in a similar pattern for each channel. The width of the interval indicates standard errors. Even controlling for variation in age, sex, STAI score, and BDI score, significant differences showed in T3 and T4. *p < 0.05. STAI-S: State-Trait Anxiety Inventory, BDI: Beck Depression Inventory, ANCOVA: analysis of covariance.

The TBR was obtained by the dividing theta wave by the beta wave, which served as an index for TCD. As previously mentioned, TBR is known to be associated with attention problems and phobic reactions^18^. When comparing the differences in TBR at each site between the two groups, the values were generally higher in TRS group than in TRP group, and this difference was particularly noticeable in the T3 (*p* = 0.036) and C3 (*p* = 0.033) areas. After controlling for variation, significant differences were observed in the T3 (*p* = 0.037) and T4 (*p* = 0.044) regions (Fig. 2).

## Discussion

In this study, patients diagnosed with anxiety disorder using DSM-5 and prescribed medications, were classified into TRS and TRP groups based on their drug response after 8–12 weeks of treatment. qEEG characteristics were investigated, and the findings confirmed that qEEG has value as a marker for identifying responders to antidepressant therapy among patients with anxiety disorders.

There were no differences in the demographic characteristics of the study participants in terms of age and sex. In addition, the two groups showed no significant difference in STAI, BDI, or SCL-90-R scores, other than in the HOS subscale score, indicating that subjective symptoms such as depression and anxiety were not significantly different between the two groups. However, the CGI-S score showed a rather significant difference but with no significant difference in the medications used, indicating that the clinician judged that the disease was more serious at the first interview for patients in the TRP group.

We recorded the patient's EEG in the eye-closed resting state for 5 min and acquired absolute power through power spectrum analysis. Higher beta waves appeared at T3 and T4 in the TRP group than in the TRS group. Notably, after controlling for variations in sex, age, STAI-S, STAI-T, and BDI scores, the differences in T3 and T4 were more consistent than before.

To the best of our knowledge, no previous studies have evaluated the treatment response in patients with anxiety disorders using qEEG, although one case report reported a reduction in overall beta waves as treatment progressed in anxiety disorders^19^. Despite the limited number of studies on treatment response, many studies have explored the qEEG characteristics of anxiety disorders. In particular, increases in beta and high-beta waves in both temporal lobes are well recognized, which have also been associated with fear, panic, insecurity, and phobia^12,15^. Beta waves in T3 and T4 are especially known to be associated with excessive amygdala activity^20^. The amygdala acts like a fear sensor that signals the hypothalamus to secrete corticotropin releasing hormone(CRH) and produce adrenocorticotropic hormone(ACTH)^18^. In addition, beta waves reflect a significant level of local metabolic activity in the cortex, and TBR elevation in anxiety disorders is also well known^13,16^. Elevated TBR suggests a disturbance in the thalamus-cortex interaction and shows a negative correlation with attentional control and trait anxiety^17^.

There are two possible explanations for the results. First, there may have been differences in anxiety symptom severity between the two groups. STAI-S and STAI-T were performed before the start of treatment and showed no significant differences, but the CGI-S evaluated by the clinician differed significantly between groups. In fact, existing studies have repeatedly reported that the more severe the symptoms of anxiety disorder, the higher the probability of responding to treatment^21^. Second, the TRS group may have included patients with comorbid diseases other than anxiety disorder. In fact, anxiety is a common symptom that accompanies depressive disorder and is often comorbid with depression^22^. However, we considered that the two groups showed biological differences since there was no significant difference in the BDI scores between the two groups, and the difference in T3 and T4 remained, even after adjusting for BDI, STAI-S, and STAI-T scores. Anxiety disorder is a heterogeneous disease that includes several diseases, and its pathophysiology has not yet been clearly elucidated; however, the above-mentioned abnormalities in the amygdala circuit and Hypothalamic–Pituitary–Adrenal (HPA) axis have been established as the main causes^23^. In this study, the TRP group showed higher beta waves in T3 and T4 compared with the TRS group, which is considered to be closer to the type of anxiety disorder characterized by excessive activity of amygdala^20^. On the other hand, abnormalities in the TRS group can be expected to reflect abnormalities of the Raphe nuclei or hippocampus, or other factors that can affect the HPA axis other than amygdala^24^. In addition to HPA axis abnormalities, oxidative stress in the central nerve system (CNS) has also been shown to cause anxiety disorder. Therefore, this group of patients may also require antioxidants in addition to the usual antidepressant treatment^25^.

This study has some limitations. The primary limitation is that the study was conducted by retrospectively reviewing medical records. Additional tests could therefore not be performed. Future follow-up studies should include other prospective assessments, including an initial evaluation and EEG after 8–12 weeks. Second, the number of study participants was small due to the limitations of the retrospective study. Several previous studies comparing the qEEG characteristics of treatment responsiveness in other diseases used a relatively small sample size to confirm treatment responsiveness^26^. However, although detailed analysis was attempted to determine the impact on other characteristics (such as sex), it could not be performed due to the small number of study participants. Third, only CGI-S was used to determine the therapeutic effect. As a result, assessment of the treatment response was based only on the clinician's judgment. Subjective evaluation of patients would have been possible if an STAI assessment, which was performed at the beginning of treatment, was also performed after treatment. Lastly, this study evaluated treatment response by recruiting a patient group from a broad category of anxiety disorders. Therefore, future studies should explore the quantitative EEG characteristics for treatment response according to each disease group.

## Conclusion

In clinical practice, qEEG is used as an auxiliary tool in the treatment of anxiety disorders, and is gradually proving its usefulness. In addition to diagnosing anxiety disorders, predicting their reactivity prior to treatment is also important, and qEEG has shown promise in this regard. Patients with anxiety disorders who show elevated T3 and T4 and decreased TBR in beta waves were more likely to respond to drug treatment using antidepressants and benzodiazepines. For patients with anxiety disorders who do not show such characteristics, combination with agents such as antioxidants or other non-pharmaceutical treatment modalities may be preferable.

## Methods

### Participants

A retrospective study based on medical records was conducted by selecting patients who underwent outpatient treatment for anxiety disorders at the Department of Psychiatry at Daegu Catholic University Hospital from March 01, 2016 to December 31, 2020. Before starting treatment, the patients underwent a qEEG examination, and they subsequently received drug treatment for more than 8 weeks. Patients who had been previously diagnosed with neurological conditions, such as convulsive disorders, had undergone intracranial surgery or insertion of magnetic material into the head or eyeball, had not received antidepressants for the first treatment, or showed poor-quality EEG findings were excluded from the study. In accordance with a previous report, patients with a CGI-S score of ≥ 4 after 8 weeks of sufficient antidepressant treatment were classified as treatment resistant (TRS group), and those with a CGI-S score of ≤ 3 were classified as treatment responsive (TRP group)^27^.

### Informed consent

This study was approved by the Institutional Review Board (IRB) of the Daegu Catholic University Medical Center (DCUMC IRB approval No. CR-21-121) and was performed in accordance with the Declaration of Helsinki (World Medical Association: Ethical Principles for Medical Research Involving Human Subjects, 1964). The IRB of the Daegu Catholic University Medical Center approved the waiver for informed consent because this was a retrospective study.

### Detailed methods

A total of 86 patients who had undergone diagnoses of the category of anxiety disorders, including generalized anxiety disorder, social anxiety disorder, and panic disorder, by a psychiatrist using the 5th edition of the Diagnostic and Statistical Manual of Mental Disorders (DSM-5) during the study period were included. The patient’s medical records were checked to determine their sex, age, type and dose of prescription drugs, and status from the start of treatment to 8 or 12 weeks. Assessments based on the STAI, BDI, and SCL-90-R were performed, and the CGI-S scale was used for disease severity assessment by clinicians. To analyze the dose of the prescribed drug, it was converted into an equivalent dose. For antidepressants, fluoxetine was used as the standard, and for benzodiazepines, diazepam was used as the standard^28,29^. Finally, the results of qEEG examinations performed before starting drug treatment were analyzed.

## Measures

### State-trait anxiety inventory

State-Trait Anxiety Inventory (STAI) is a self-reporting tool that has been proven to be useful for measuring anxiety in the general population and clinical samples. It consists of two subscales: the “State,” which evaluates the current state, and the “Trait,” which evaluates the general state. Each subscale includes 20 items that are evaluated on a 4-point Likert scale of 1–4 points, and a score of ≥ 40 is considered to be clinically significant. The internal consistency coefficient of the subscales was 0.86–0.95 and the test repeatability coefficient was 0.65–0.75 for 2 months. The composition and validity of the test are widely known^30^.

### Beck depression inventory

The Beck depression inventory (BDI) is a self-reported test developed by Beck et al.^31^ and is the most commonly used tool to measure depressive symptoms. It consists of a total of 21 items that are rated on a 4-point Likert scale with 0–3 points. This scale was used to evaluate depressive symptoms that may be associated with anxiety.

### Symptom checklist-90-revised

The symptom checklist-90-revised (SCL-90-R) is a self-reported test and is commonly used to measure overall psychiatric symptoms. The test evaluates somatization (SOM; 12 items), obsessive–compulsive characteristics (OC; 10 items), interpersonal sensitivity (IS; 9 items), depression (DEP; 13 items), anxiety (ANX; 10 items), hostility (HOS; 6 items), phobic anxiety (PHOB; 7 items), paranoid ideation, (PAR; 6 items), and psychoticism (PSY; 10 items) using symptom-level detailed scales. Each item is scored on a 4-point Likert scale consisting of 0–3 points. This scale was used to evaluate the patient's other psychiatric symptoms^32^.

### Clinical global impression-severity

As one of the most commonly used evaluation tools in the field of psychiatry, the clinical global impression-severity (CGI-S) scale can be used to directly evaluate patients by measuring the severity of symptoms, cure rates, and treatment effectiveness. It consists of a score from 1 to 7. The severity of the patient's symptoms was evaluated according to standardized scoring guidelines^33^.

### EEG recording and pre-processing

For EEG measurement, 19 channels of the international 10–20 system (Fp1, Fp2, F7, F3, Fz, F4, F8, T3, C3, Cz, C4, T4, P7, P3, Pz, P4, P8, O1, O2) were used, and a 64-channel Comet digital EEG unit (Grass, Natus neurology, USA) was used for measurements with an ear electrode recording frequency of 800 Hz. EEG measurements were obtained for 5 min whilst the patient was lying on a comfortable bed with their eyes open, and then for 5 min with their eyes closed immediately afterwards. The patients were instructed to keep an eye on the “ + ” sign in the front of them when their eyes were open, to refrain from movement as much as possible, to remain in a state of not thinking about anything as much as possible, and to not fall asleep when the eyes were closed. EEG analysis was performed using the fast Fourier transforms (FFT) algorithm for each frequency band for each selected epoch: delta wave (1–4 Hz), theta wave (4–8 Hz), alpha wave (8–12 Hz), and beta wave (12–30 Hz). Beta waves were subdivided into low-beta (12–15 Hz), beta (15–25 Hz), and high-beta (25–30 Hz)^14^. MATLAB 7.0.1 (MathWorks, Massachusetts, U.S.A) and EEGLAB toolbox were used for analysis. To calculate the TBR, the theta wave was divided by the beta wave and used for analysis. For analysis, down-sampling of EEG data to 250 Hz, detrending, and mean-subtracting were performed to remove the DC component. Then, frequencies ≤ 1 Hz and ≥ 60 Hz that were affected by electrical noise were removed through the filter, and the noise caused by blinking and muscle movement was then removed through independent component analysis (ICA). Finally, clinical psychiatrists and EEG experts visually inspected the corrected EEGs. For the analysis, we selected more than 2 min of artifact-free EEG readings from the five 3-min recordings.

### Statistical analysis

The Student t-test was used to compare continuous sociodemographic variables between the two groups (TRS and TRP), and the chi-square test was used for comparison of categorical variables. For each frequency and TBR at each site between the two groups, the total score of sex; age; and BDI, STAI-S, and STAI-T scores was set as a covariate, and then the difference between groups was analyzed using ANCOVA. Equality of variance was tested using Levene's test. Significance was set at a p-value of 0.05, and all analyses were performed using SPSS Version 25.0 for Windows (IBM Corp., Armonk, NY).

## Supplementary Information

Supplementary Information.

## Data Availability

The datasets used and analyzed in this study are available from the corresponding author upon reasonable request.
