# Supplementary material for: Quantitative electroencephalographic biomarker of pharmacological treatment response in patients with anxiety disorder: a retrospective study
Source: Sci Rep. 2023 Mar 7;13:3802. doi: 10.1038/s41598-023-30994-1 (PMC9992385; doi:10.1038/s41598-023-30994-1)
Supplement: Supplementary file 1 — Supplementary Information. [file 41598_2023_30994_MOESM1_ESM.docx]

**Supplement 1. Clinical characteristics of Subjects**

| Characteristics | Total subjects (n=86) | Treatment resistant anxiety disorder  (n=56) | Treatment response anxiety disorder  (n=30) |
| --- | --- | --- | --- |
| Diagnosis, n(%) |  |  |  |
| Generalized anxiety disorder | 72 (83.7%) | 45 (80.4%) | 27 (90.0%) |
| Panic disorder | 12 (14.0%) | 9 (16.1%) | 3 (10.0%) |
| Social anxiety disorder | 2 (2.3%) | 2 (3.6%) | 0 (0.0%) |
| Comorbidity, n(%) | 26 (30.2%) | 14 (75.0%) | 12 (60.0%) |
| Major depressive disorder | 12 (14.0%) | 5 (8.9%) | 7 (23.3%) |
| Somatic symptom disorder | 9 (10.5%) | 4 (7.1%) | 5 (16.7%) |
| Obsessive-compulsive disorder | 3 (3.5%) | 3 (5.4%) | 0 (0.0%) |
| Alcohol use disorder | 2 (2.3%) | 2 (3.6%) | 0 (0.0%) |
| Antidepressant(initial), n(%) |  |  |  |
| Escitalopram | 49 (57.0%) | 33 (58.9%) | 16 (53.3%) |
| Paroxetine | 11 (12.8%) | 6 (10.7%) | 5 (16.7%) |
| Sertraline | 7 (8.1%) | 3 (5.4%) | 4 (13.3%) |
| Vortioxetine | 7 (8.1%) | 5 (8.9%) | 2 (6.7%) |
| Desvenlafaxine | 6 (7.0%) | 4 (7.1%) | 2 (6.7%) |
| Mirtazapine | 6 (7.0%) | 5 (8.9%) | 1 (3.3%) |
|  |  |  |  |
